# 1219. Utility of Plasma Microbial Cell-Free DNA Testing for Therapeutic Management and Antimicrobial Stewardship

**DOI:** 10.1093/ofid/ofad500.1059

**Published:** 2023-11-27

**Authors:** Riley Thompson, Monica Donnelley, George R Thompson, Angel Desai

**Affiliations:** University of California Davis Health, Sacramento, California; University of California Davis Medical Center, Sacramento, California; University of California Davis Medical Center, Sacramento, California; University of California Davis Medical Center, Sacramento, California

## Abstract

**Background:**

Microbial cell-free DNA (mcfDNA) sequencing is a promising potential tool in the detection of infectious pathogens when traditional methods fail to identify the causative agent. It may be particularly helpful in identifying pathogens that are present at low levels or that do not grow well in standard culture. The aim of this study was to determine whether plasma mcfDNA sequencing resulted in a change in therapeutic management.

**Methods:**

A retrospective cohort at UC Davis Medical Center was reviewed. All patients, both pediatric and adult, that underwent plasma mcfDNA (Karius) sequencing between December 11^th^, 2019, and January 4^th^, 2023, were included. Patient demographics, comorbid conditions, immunocompromised status, indication for testing, pre and post antimicrobial therapies from when the sample was collected, pathogen identified, ID consultation status, and ordering service at the time of testing were recorded. Each patient was analyzed by two independent reviewers for clinically significant microbial findings, treatment, and reviewer concordance with therapeutic management. If discordance between reviewers was present, a third reviewer analyzed the data.

**Results:**

A total of 127 Karius tests were reviewed from 71 patients. Baseline characteristics included 62 (48%) pediatric and 65 (51%) adult tests. 25 (33%) tests were from female patients, and 37 tests (50%) were obtained from immunocompromised patients. The most frequent adult ordering services included the Medical Intensive Care Unit and Bone Marrow Transplant Unit. The most frequent pediatric ordering services included the Pediatric Intensive Care Unit followed by General Pediatrics. 69 (95%) of tests had a formal Infectious Diseases consultation associated with testing. 22 (30%) tests resulted in a change in antimicrobial therapy, and 35 (47%) of tests resulted in a clinically significant organism.

**Conclusion:**

In this retrospective cohort study conducted at a single center, Karius testing resulted in clinically significant pathogens in less than half of all tests conducted, and most test results did not lead to a change in therapeutic management. Testing in certain clinical scenarios or high-risk settings may be warranted, however further studies are needed.

**Disclosures:**

**George R. Thompson, III, MD**, Astellas: Advisor/Consultant|Astellas: Grant/Research Support|Cidara: Advisor/Consultant|Cidara: Grant/Research Support|F2G: Advisor/Consultant|F2G: Grant/Research Support|Mayne: Advisor/Consultant|Mayne: Grant/Research Support|Melinta: Advisor/Consultant|Melinta: Grant/Research Support|Mundipharma: Advisor/Consultant|Mundipharma: Grant/Research Support|Pfizer: Advisor/Consultant|Pfizer: Grant/Research Support

